# A Subset of Cerebrospinal Fluid Proteins from a Multi-Analyte Panel Associated with Brain Atrophy, Disease Classification and Prediction in Alzheimer’s Disease

**DOI:** 10.1371/journal.pone.0134368

**Published:** 2015-08-18

**Authors:** Wasim Khan, Carlos Aguilar, Steven J. Kiddle, Orla Doyle, Madhav Thambisetty, Sebastian Muehlboeck, Martina Sattlecker, Stephen Newhouse, Simon Lovestone, Richard Dobson, Vincent Giampietro, Eric Westman, Andrew Simmons, Alzheimer’s Disease Neuroimaging Initiative

**Affiliations:** 1 King’s College London, Institute of Psychiatry, Psychology & Neuroscience, London, United Kingdom; 2 NIHR Biomedical Research Centre for Mental Health, London, United Kingdom; 3 NIHR Biomedical Research Unit for Dementia, London, United Kingdom; 4 Department of Neurobiology, Care Sciences and Society, Karolinska Institutet, Stockholm, Sweden; 5 Laboratory of Behavioural Neuroscience, National Institute on Aging, National Institutes of Health, Baltimore, Maryland, United States of America; 6 Department of Psychiatry, University of Oxford, Oxford, United Kingdom; Nathan Kline Institute and New York University School of Medicine, UNITED STATES

## Abstract

In this exploratory neuroimaging-proteomic study, we aimed to identify CSF proteins associated with AD and test their prognostic ability for disease classification and MCI to AD conversion prediction. Our study sample consisted of 295 subjects with CSF multi-analyte panel data and MRI at baseline downloaded from ADNI. Firstly, we tested the statistical effects of CSF proteins (n = 83) to measures of brain atrophy, CSF biomarkers, ApoE genotype and cognitive decline. We found that several proteins (primarily CgA and FABP) were related to either brain atrophy or CSF biomarkers. In relation to ApoE genotype, a unique biochemical profile characterised by low CSF levels of Apo E was evident in ε4 carriers compared to ε3 carriers. In an exploratory analysis, 3/83 proteins (SGOT, MCP-1, IL6r) were also found to be mildly associated with cognitive decline in MCI subjects over a 4-year period. Future studies are warranted to establish the validity of these proteins as prognostic factors for cognitive decline. For disease classification, a subset of proteins (n = 24) combined with MRI measurements and CSF biomarkers achieved an accuracy of 95.1% (Sensitivity 87.7%; Specificity 94.3%; AUC 0.95) and accurately detected 94.1% of MCI subjects progressing to AD at 12 months. The subset of proteins included FABP, CgA, MMP-2, and PPP as strong predictors in the model. Our findings suggest that the marker of panel of proteins identified here may be important candidates for improving the earlier detection of AD. Further targeted proteomic and longitudinal studies would be required to validate these findings with more generalisability.

## Introduction

Alzheimer’s disease (AD) is a progressive neurodegenerative disorder pathologically characterised by lesions of misfolded proteins, the loss of synapses and an overall reduction in brain volume. There is accumulating evidence to suggest that the clinical symptoms of the disease are preceded by a long presymptomatic phase (~15–20 years) of abnormal β-amyloid (Aβ) aggregation in the form of extracellular senile plaques [1,2]. The neuropathology of the disease is associated with the development of neurofibrillary tangles prior to the onset of cognitive impairment and the subsequent emergence of full-blown dementia [3,4]. The failure of several clinical trials assessing therapeutic strategies to target amyloid deposition has led to the impetus to discover biomarkers earlier in the AD pathological cascade prior to the development of cognitive symptoms.

One method is to study structural neuroimaging biomarkers of AD which have been advocated for use in early diagnosis [5], as well as for predicting disease progression in a prodromal form of the disease known as Mild Cognitive Impairment (MCI) [6]. Another rich source of biomarkers can be found in analytes from cerebrospinal fluid (CSF), particularly, concentrations of Aβ142, p-tau181 and t-tau which reflect biochemical changes associated with Aβ deposition, neurofibrillary tangle formation, and neuronal cell death [7,8].

Several neuroimaging studies have since found that the combined use of MRI measures from regions affected in AD and CSF biomarkers can provide mutually complimentary information for disease classification and prediction [9,10]. Nevertheless, there still remains a substantial overlap in CSF biomarker concentrations between AD and cognitively normal (CN) individuals with an increased risk of developing the disease [11]. Moreover, additional biomarkers are still required to understand the exact temporospatial relationship between Aβ deposition and tau neurodegeneration during different stages of the disease pathophysiology. Early genetic and in-vivo experimental studies have suggested that markers of inflammation, microglial activity and synaptic function may be important for reflecting biochemical changes associated with the Aβ toxicity and tau neurodegeneration [12,13]. While some proteomic studies using multiplex platforms have identified a number of protein candidates detected in AD [14–16], few have been validated and tested in relation to well-established neuroimaging endophenotypes of AD pathology. Discovering proteins in relation to established measures of disease pathology may yield biologically important peripheral signatures associated with mechanisms early in the disease.

In this study we aimed to discover CSF proteins associated with AD pathophysiology by testing the multiplex panel with established neuroimaging measures, CSF biomarkers of AD, Apolipoprotein E (ApoE) genotype and cognitive decline. Most importantly, we aimed to identify a subset of proteins from the multiplex panel in order to test its diagnostic utility with existing AD biomarkers for disease classification and MCI to AD conversion prediction at follow up.

## Materials and Methods

### Participants

Data used in this study was obtained from the ADNI database (adni.loni.ucla.edu). ADNI was launched by the National Institute of Ageing (NIA) and is a multicenter project supported by private pharmaceutical companies, and non-profit organisations for the development of biomarkers in monitoring disease progression in MCI and AD [17]. ADNI subjects aged 55–90 from over 50 sites across the U.S and Canada participated in the research (for further information, see www.adni-info.org). Written informed consent was given from all participants in the study and prior ethics committee approval was obtained from each participating site. A total of 295 subjects with baseline data that included structural imaging and multiplex CSF samples were available for analysis and consisted of 142 subjects with MCI, 65 patients with AD, and 88 healthy control subjects.

### CSF protein measurements

CSF Aβ_1–42_, T-tau and P-tau were measured at the ADNI Biomarker Core laboratory at the University of Pennsylvania Medical Center, using the multiplex xMAP Luminex platform (Luminex, Austin, TX, USA) with the INNOBIA AlzBio3 kit (Innogenetics, Ghent, Belgium) [18,19].

CSF multiplex proteomic samples were measured for levels of 159 analytes using the Human Discovery Multi-Analyte Profile (MAP) 1.0 panel and Luminex 100 platform developed by Rules Based Medicine, Inc. (RBM), (Austin, TX) [20]. This panel is based upon multiplex immunoassay technology to measure a range of inflammatory, metabolic, lipid, and other disease relevant proteins. The protocol used to quantify CSF analytes is described in detail elsewhere [21,22]. Of the 159 analytes, only those with <10% of missing values were quantifiable leaving 83 in total for analysis. The remaining 76 analytes were mostly below the assay detection limit, or had other assay limitations. Each analyte has an individual standard curve with between 6–8 reference standards. Each plate is run with 3 levels of QCs (low, medium and high) for each analyte. A total of 16 of the CSF samples were retested using a separate never before thawed replicate aliquot on the fifth of the five 96 well plates to provide blinded test/re-test quality control data. Assays are qualified based on least detectable dose, precision, cross-reactivity, dilutional linearity, spike recovery (assessment of accuracy), and test/re-test performance. Cross validation to alternative methods is reported for some assays where feasible. Further information on the process, aliquoting and storage of analytes is described in the ADNI Biomarker Core Laboratory Standard Operating Procedures (http://adni.loni.usc.edu/wp-content/uploads/2012/01/2011Dec28-Biomarkers-Consortium-Data-Primer-FINAL1.pdf). Further assay documentation and validation reports are available from Myriad RBM (www.myriadrbm.com). Distributions of data for individual CSF proteins were checked for normality using Box-Cox methods and, when appropriate, transformed to approximate a normal distribution. Information regarding the biological preparation of CSF samples and quality control criteria of the RBM Human Discovery MAP panel can be found on the ADNI websites [23,24]. A complete list of the analytes is given in S1 Table.

### Magnetic Resonance Imaging Data Acquisition and Analysis

Structural MRI images (at 1.5T) were acquired at multiple ADNI sites across the US and Canada based on a standardized protocol [25]. The imaging protocol included a high resolution sagittal 3D T1-weighted MPRAGE volume (voxel size 1.1 × 1.1 × 1.2 mm³). The MPRAGE volume was acquired using a custom pulse sequence specifically designed for the ADNI study to ensure compatibility across scanners [26]. Full brain and skull coverage was required for all MR images according to previously published quality control criteria [27,28].

Image analysis was carried out using the Freesurfer image analysis pipeline (version 5.1.0) to produce 34 regional cortical thickness and 23 subcortical volumetric measures as previously described [29,30]. All volumetric measures from each subject were normalized by the subject’s intracranial volume while cortical thickness measures were used in their raw form [31]. Measures of hippocampal and entorhinal cortex volume were selected as key *a priori* regions to reflect AD pathology. A previously validated MRI-based marker of AD and MCI known as SPARE-AD (Spatial Pattern of Abnormalities for Recognition of Early AD) was also used as a neuroimaging marker of AD. Individualised scores of diagnostic and predictive value were used for analysis. A complete list of regional MRI measures is given in S2 Table. Details of this particular method have been widely published elsewhere [9,32,33].

### Statistical Analysis

Firstly, the RBM panel of CSF proteins were tested in relation to regional MRI measurements (hippocampal and entorhinal volume), an MRI-based measure known as SPARE-AD score and CSF biomarkers (Aβ142, P-tau_181_, T-tau) using a Spearman rank partial correlation test. This test was adjusted for covariates including age, gender, years of education and ApoE E4 genotype. Secondly, CSF proteins from the RBM panel were also tested in relation to different ApoE polymorphisms (ε2 carriers, ε3 carriers and ε4 carriers) using a generalized linear model adjusting for age, gender and years of education. Thirdly, to test the effect of CSF proteins on longitudinal MMSE score, we used a linear mixed model approach. Global MMSE score was used as the response variable and the time from baseline visit in months, CSF protein from the RBM panel, age, sex, years of education and ApoE ε4 genotype were included as fixed effects. Models contained a random intercept and slope. The applicability of our mixed models were assessed by examining models with and without the random effect of data collection site, the linearity of CSF proteins over time within subjects and the normality of model residuals using diagnostic plots. All models were tested in both AD patients (n = 59) and MCI subjects (n = 142) with serial MMSE measurements. As a large number of proteins from the RBM panel were tested we used a false discovery rate correction to account for multiple comparisons.

A multivariate support vector machine (SVM) algorithm was applied to the ADNI cohort, in an unbiased fashion, to distinguish AD patients from CN individuals. In particular, a linear SVM algorithm was constructed using the LIBSVM implementation [34]. In the algorithm, the parameter C (representing the error/trade off parameter used for adjusting separation error in the creation of separation space) was optimized using 5-fold cross validation on the training set. The grid search routine suggested by Hsu et al (2010) [35] was implemented to identify optimal parameter settings for differentiating AD from CN individuals. A multi-kernel learning approach for linear SVM [36] was implemented for treating variables of a different nature. A general framework for kernel methods used to integrate data from different modalities has been described previously in more extensive detail [36–38].

To identify a subset of CSF proteins associated with AD, we adopted a recursive feature elimination (RFE) wrapper. The final subset of CSF proteins (CSF RFE subset) were then combined with CSF biomarkers and regional MRI measurements to test their utility for disease classification. Classification accuracy in each of these models was evaluated using ten-fold cross validation. Measures of accuracy, sensitivity, specificity, and area under the curve (AUC) were used to compare AD vs. CN models.

MCI subjects were divided into subjects that progressed to an AD diagnosis (MCI-converters) and others that remained clinically stable over a 12 month follow up period (MCI non-converters). Subsequently, models from the AD vs. CN comparisons were used as training classifiers to prospectively predict MCI to AD conversion in MCI converters (MCI-c), as well as predicting MCI non-converters (MCI-nc) that remained stable at 12 months. Discriminant scores from the model were then used to classify MCI subjects as either having an AD-like or CN-like phenotype. The combined model (CSF RFE subset + CSF biomarkers + regional MRI measures) was also used to predict MCI to AD conversion in moderately late MCI-c (subjects that progressed to AD between 18–24 months follow up) and late MCI-c (subjects that progressed to AD at 36 months). MCI-nc predictions were also made using the combined model for subjects that remained clinically stable between 0–12 months, 18–24 months and 36 months follow up.

The R statistical software environment (v. 3.1.0; The R Foundation for Statistical Computing), was used to perform all statistical analyses.

## Results

### Demographic Characteristics

Baseline sample characteristics of the ADNI cohort for demographic, cognitive, MRI and CSF biomarkers are presented by diagnostic group in Table 1. Significant differences between groups were found in hippocampal and entorhinal volume, as well as, SPARE-AD score, CSF biomarkers of AD, MMSE score and ApoE ε4 genotype. Subject age, gender and years of education were not found to differ significantly between groups.

**Table 1:** Demographic characteristics of the ADNI cohort.

	AD (*n* = 65)	MCI (*n* = 142)	CN (*n* = 88)	*p*-value
Age	74.6 ± 7.6	74.9 ± 7.3	75.8 ± 5.5	0.491
Gender (male/female)	29/36	47/95	44/46	0.061
Education (years)	15.9 ± 3.1	15.5 ± 3.1	15.5 ± 2.9	0.560
MMSE score	23.5 ± 1.9^a^ ^,^ ^b^	27.1 ± 1.7^b^	29.1 ± 1.0	<0.001
ApoE ε4 genotype (+ve/-ve)	46/19	76/66	22/66	<0.001
Hippocampal Volume (mL)	1.82 ± 0.4^b^	1.94 ± 0.3^b^	2.34 ± 0.3	<0.001
Entorhinal Volume (mL)	0.95 ± 0.2^a^ ^,^ ^b^	1.12 ± 0.2^b^	1.28 ± 0.2	<0.001
ICV (mL)	1314 ± 158	1328 ± 139	1290 ± 136	0.146
SPARE-AD score	1.21 ± 0.8^a^ ^,^ ^b^	0.81 ± 0.8^b^	-1.5 ± 0.9	<0.001
Aβ1–42	140.4 ± 35.3^a^ ^,^ ^b^	159.6 ± 51.7^b^	205.7 ± 57.2	<0.001
t-tau	125.9 ± 60.3^a^ ^,^ ^b^	104.8 ± 52.5^b^	69.2 ± 27.9	<0.001
p-tau181	42.2 ± 20.7^b^	36.5 ± 16.1^b^	24.9 ± 13.2	<0.001

Data are represented as mean ± and standard deviation. AD = Alzheimer’s disease, MCI = Mild Cognitive Impairment, CN = cognitively normal individuals, MMSE = Mini Mental State Examination, ICV = Intracranial Volume, SPARE-AD score = Spatial Pattern of Abnormalities for Recognition of Early AD. Chi-square was used for gender and ApoE ε4 genotype comparison. One way ANOVA with Bonferroni post hoc test was used for continuous measures.

^a^ indicates significant compared to the MCI group.

^b^ indicates significant compared to the CN group.

### CSF proteins from the multiplex RBM panel associated with neuroimaging markers of brain atrophy and CSF biomarkers of AD

**Fig 1:**
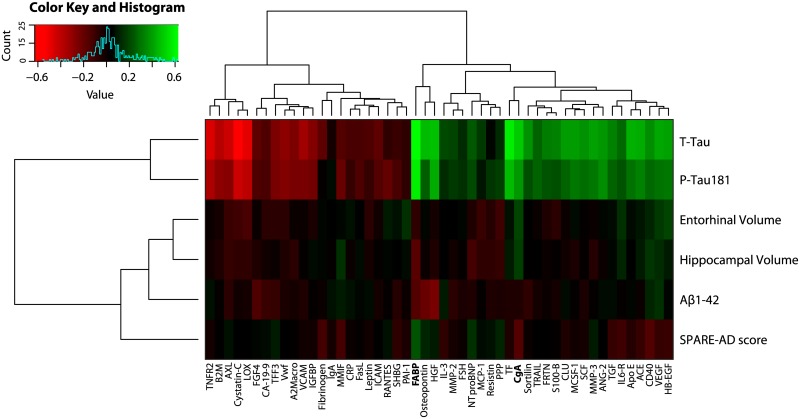
Heatmap of baseline CSF proteins that were significantly associated with regional MRI measures, SPARE-AD score or CSF biomarkers in AD patients and MCI subjects (n = 207).

Due to the exploratory nature of this study we first tested the association of the entire multiplex panel of CSF proteins (n = 83) with neuroimaging and CSF biomarkers to identify candidates related AD pathogenesis. Associations were tested using a partial spearman rank correlation test that co-varied for the effects of age, gender, years of education and ApoE ε4 genotype. For several proteins (n = 50) we found an association with either neuroimaging markers of brain atrophy or CSF biomarkers of AD (Fig 1). Many proteins in this subset (n = 37) were also found to be significantly associated with both P-tau181 and T-tau CSF levels. Reduced levels of CgA were found to be significantly associated across all comparisons with neuroimaging and CSF biomarkers, but only remained significant in association with hippocampal (*p* = <0.001)and entorhinal volume (*p* = 0.008) after multiple comparison correction. Increased levels of Fatty Acid Binding Protein (FABP) emerged as the most significantly associated with CSF levels of P-tau181 and T-tau as well as SPARE-AD score. Results from our partial spearman rank correlation test are displayed in S3 Table. Bear in mind most associations were mild and reflected by p-values that were uncorrected for multiple comparisons.

### CSF proteins associated with different ApoE gene polymorphisms

**Fig 2:**
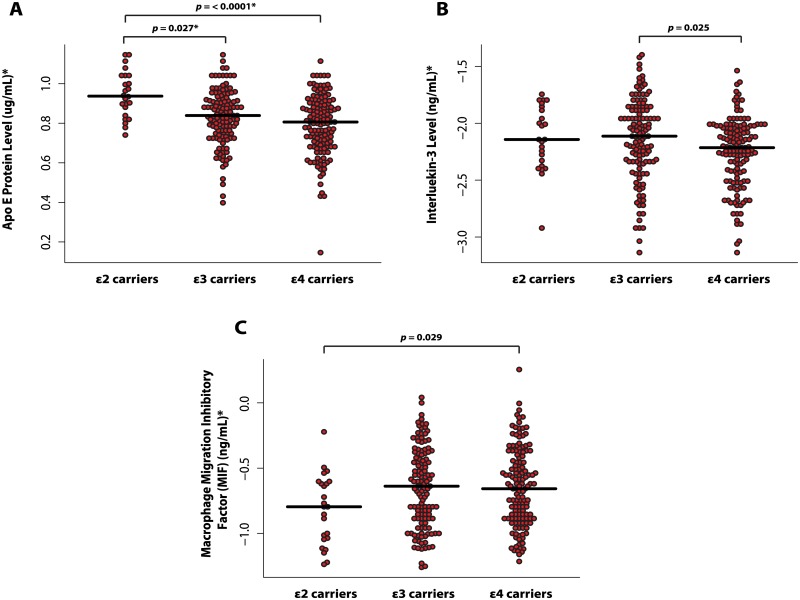
CSF proteins significantly associated with different ApoE gene polymorphisms (ε2 carriers, ε3 carriers, and ε4 carriers). **(A)** CSF levels of ApoE protein between ApoE groups; **(B)** CSF levels of Interleukin-3 (IL-3) between ApoE groups and **(C)** CSF levels of Macrophage migration inhibitory factor (MIF) between ApoE groups. *These units refer to data before transformation.

CSF proteins from the RBM panel were also tested in relation to the ApoE polymorphism rather than diagnostic status. Significant differences in CSF levels were examined by ApoE genotype (ε2 carriers, ε3 carriers and ε4 carriers). We found that 9 CSF proteins were associated with the overall effect of ApoE genotype (Apo E, FABP, FGF-4, IL-8, AGRP, MIF, IL-3, ANG-2, and Osteopontin). However, only CSF levels of Apo E, IL-3 and MIF were found to differ between ApoE groups. CSF levels of these proteins compared to each ApoE group are shown in Fig 2. In particular, the strongest overall effect was observed for CSF levels of Apo- E which passed multiple comparison correction (*p* = .00046; FDR corrected = .034). Pairwise comparisons revealed that Apo-E levels were significantly lower in ε4 carriers irrespective of diagnosis compared to ε2 carriers (*t* = -3.63; *p* = < .0001) and significantly lower in ε3 carriers compared to ε2 carriers (*t* = -2.57; *p* = .027).

### CSF proteins related to the rate of cognitive decline on longitudinal MMSE score

We also tested the association of baseline CSF proteins with the rate of cognitive decline using at least three or four serial measurements of MMSE score. Firstly, we tested this in a sample of AD patients (*n* = 59) and found no CSF proteins were able to significantly predict a longitudinal change in MMSE score. However, in a sample of MCI subjects (*n* = 142) we found three proteins (SGOT, MCP-1, and IL-6r) were able to significantly predict cognitive decline (Table 2). Again, it should be noted that these associations were mild and no protein remained significant after multiple comparison correction.

**Table 2:** CSF proteins significantly predicting a longitudinal decline on MMSE score in a sample of MCI subjects (*n* = 142).

	Linear mixed effect models		
CSF protein	β	*S*.*E*	*P*-value
Serum Glutamic Oxaloacetic Transaminase (SGOT)	0.34	0.13	0.0074
Monocyte Chemotactic Protein 1 (MCP-1)	-0.21	0.09	0.027
Interleukin-6 receptor (IL-6r)	0.20	0.08	0.022

Linear mixed effect model results are displayed as on b-coefficients (β), standard-error

(*S*.*E*) and P-values for the interaction terms between proteins and time (years from baseline).

Data were adjusted for age, gender, years of education and ApoE genotype as fixed effects and subject code and site-id as random effects.

### Disease Classification

**Fig 3:**
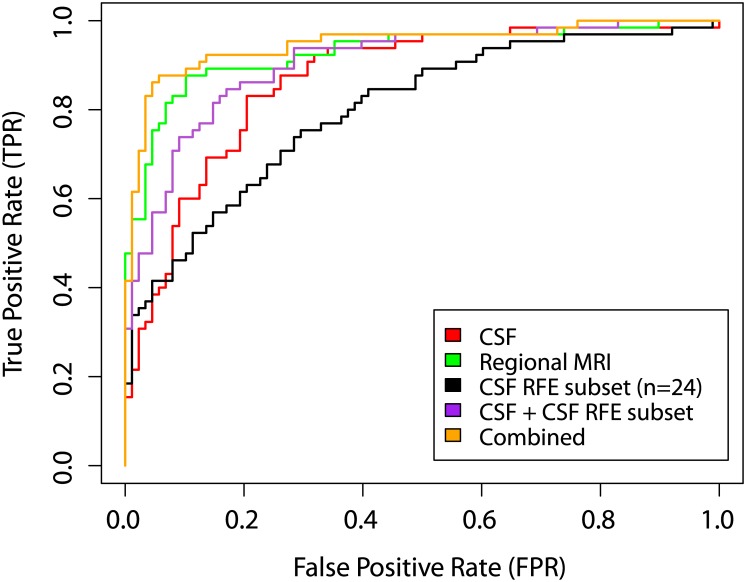
ROC curves from disease classification models for differentiating between AD and CN individuals.

The recursive feature elimination (RFE) wrapper method identified a subset of 24 CSF proteins which best distinguished AD patients from CN individuals (Table 3). Overall, we found that the inclusion of these CSF proteins from the RBM panel improved the accuracy and specificity of models. In particular, combining the CSF RFE subset with CSF biomarkers resulted in an accuracy of 84.3% and an AUC of 91% (Table 4). We found that combining the CSF RFE subset improved the sensitivity in a model generated using CSF biomarkers from 70.8% to 83.1% which was statistically significant (Venkatraman’s Test: Z = 2.94; *p* = .0042). Moreover, the CSF RFE subset combined with CSF biomarkers and regional MRI measures, achieved an accuracy of 91.5% (SEN = 87.7%, SPE = 94.3%, AUC = 0.95) which was significantly better than using CSF biomarkers alone (Z = 2.91; *p* = .0036) (Fig 3). In the combined model (CSF RFE subset + CSF biomarkers + regional MRI measures) we found CgA, FABP, MMP-2, and PPP contributed most strongly toward the detection of AD. However, the regional MRI measures and CSF biomarkers model gave the best result with an accuracy of 92.2% (SEN = 85.7%, SPE = 96.4%, AUC = 0.96) but this was not found to be significantly better than the CSF RFE subset combined with CSF biomarkers and regional MRI measures (Z = 0.38; *p* = 0.70). All model results are shown in Table 4.

**Table 3:** CSF proteins selected in the CSF RFE subset using a built-in importance measure (SVM-RFE wrapper) for differentiating AD patients from CN individuals.

Rank	CSF multi-analyte subset
1	Fatty acid binding protein (FABP)
2	Chromogranin-A (CgA)
3	Osteopontin
4	Pancreatic polypeptide (PPP)
5	Interleukin-3 (IL-3)
6	Resistin
7	Cancer Antigen 19–9 (CA-19-9)
8	Apolipoprotein E (Apo E)
9	Calcitonin
10	Hepatocyte Growth Factor (HGF)
11	Fibroblast Growth Factor 4 (FGF-4)
12	Matrix Metalloproteinase-3 (MMP-3)
13	C-Reactive Protein (CRP)
14	Adiponectin
15	AXL Receptor Tyrosine Kinase (AXL)
16	Endothelin-1 (ET-1)
17	Apolipoprotein(a) (Lp(a))
18	Pregnancy-Associated Plasma Protein A (PAPP-A)
19	CD 40 antigen (CD40)
20	Agouti-Related Protein (AGRP)
21	Myoglobin
22	Matrix Metalloproteinase-2 (MMP-2)
23	Thyroxine-Binding Globulin (TBG)
24	Plasminogen Activator Inhibitor 1 (PAI-1)

**Table 4:** Accuracy, sensitivity, specificity and area under the curve of AD vs. CN models.

	ACC (%)	SEN (%)	SPE (%)	AUC
**CSF RFE subset (n = 24)**	72.6	70.8	73.9	0.80
**CSF biomarkers**	77.1	70.8	81.8	0.87
**Regional MRI measures**	87.6	81.5	92.1	0.93
**CSF RFE subset + CSF biomarkers**	83.0	83.1	83.0	0.90
**CSF biomarkers + regional MRI measures**	92.2	85.7	96.4	0.96
**Combined**	91.5	87.7	94.3	0.95

Data are percentages and confidence intervals are presented in parenthesis.

ACC = Accuracy, SENS = sensitivity, SPE = specificity, AUC = area under the curve.

The combined model includes regional MRI measures, CSF biomarkers of AD and the CSF RFE subset of proteins (n = 24).

### MCI to AD conversion prediction

Over a follow up period of 36 months, 72 MCI subjects (50.7%) from our sample converted to an AD diagnosis. Table 5 shows the number of MCI subjects that were predicted as either AD-like or CN-like at a 12 month follow-up interval using all AD vs. CN models.

**Table 5:** MCI to AD conversion prediction at a one year follow up using the AD vs. CN multivariate models.

	MCI-c Classification (n = 34)		MCI-nc Classification (n = 108)	
	AD like (%)*	CN like (%) **	AD like (%)*	CN like (%) **
CSF RFE subset (n = 24)	**82.4 (28)**	17.6 (6)	71.3 (71)	**28.7 (31)**
CSF biomarkers	**73.5 (25)**	26.5 (9)	56.5 (61)	**43.5 (47)**
Regional MRI measures	**73.5 (25)**	26.5 (9)	42.6 (46)	**57.4 (64)**
CSF RFE subset + CSF biomarkers	**88.2 (30)**	11.8 (4)	67.6 (73)	**32.4 (35)**
CSF biomarkers + regional MRI measures	**76.5 (26)**	23.5(8)	38.9 (42)	**61.1 (66)**
Combined	**94.1 (32)**	5.9 (2)	75.0 (81)	**25.0 (27)**

AD = Alzheimer’s disease, MCI = Mild Cognitive Impairment, MCI-c = MCI converter, MCI-nc = MCI non-converter, CN = Cognitively Normal.

*Sensitivity is the percentage of MCI-c subjects correctly classified as AD in bold.

**Specificity is the percentage of MCI-nc subjects correctly classified as CN in bold.

The combined model includes regional MRI measures, CSF biomarkers of AD and the CSF RFE subset of proteins (n = 24).

**Fig 4:**
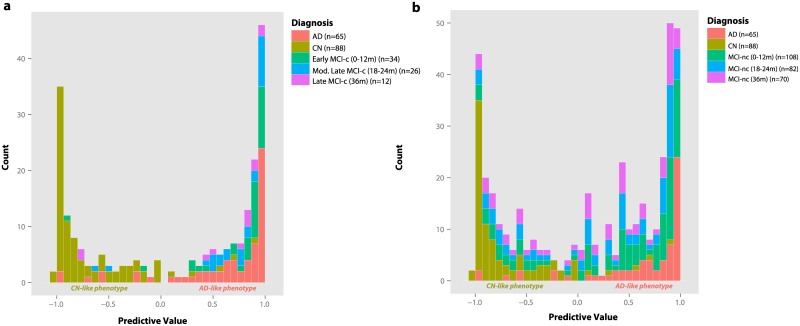
Predictive values from the combined CSF RFE subset CSF biomarker and regional MRI measures model for MCI to AD conversion prediction at several follow up timepoints. **(A)** Predictive values of MCI-c progressing to AD at different follow up timepoints overlaid with predictive values of AD and CN individuals and **(B)** predictive values of MCI-nc at different follow up timepoints overlaid with predictive values of AD and CN individuals.

Firstly, we tested all models in early MCI subjects who progressed to an AD diagnosis between 0–12 months (*n* = 34) [Early MCI-c]. We found that the inclusion of the CSF RFE subset with CSF biomarkers and regional MRI measures provided the best result, accurately predicting 94.1% of MCI-c progressing to AD whereas the regional MRI measures and CSF biomarker model was only able to achieve a prediction of 76.5%. Therefore, we further tested the combined model in moderately late MCI-c (*n* = 26) who were also correctly predicted with a 92.3% accuracy as progressing to AD, and late MCI-c (*n* = 12) who were predicted correctly with an 82.4% accuracy. Fig 4a displays the predicted probabilities from the combined model of MCI subjects that progressed to AD. For comparison, we also overlaid the predicted probabilities of AD patients and CN individuals. The majority of subjects that converted to AD at different follow up periods were found to already possess an AD-like phenotype at the prodromal MCI stage (p > 0.05; Kolmogorov-Smirnov test).

In contrast, MCI-nc predictions were less accurate with predictions ranging from 57.4% to 25.0%. Over a 36 month follow up period 70 MCI subjects (49.3%) remained clinically stable. The regional MRI measures model was found to yield the best prediction at a 12 month follow up. For the combined model, Fig 4b displays an almost bimodal distribution of MCI-nc predictive values, with some correctly predicted as CN-like and others predicted as having an AD-like phenotype. Despite these subjects remaining clinically stable at their respective period of follow up, some MCI subjects are expected to convert in the near future and as a result also display an AD-like phenotype at baseline. Further follow ups will determine whether these subjects remain clinically stable or convert to an AD diagnosis.

## Discussion

In this neuroimaging-proteomic study there were a number of key findings. Firstly, we identified several CSF proteins (n = 50) related to neuroimaging phenotypes of brain atrophy and CSF biomarkers of AD, suggesting that these candidates may be related to AD pathophysiology. Second, a unique biochemical profile of CSF proteins was found to be associated with ApoE genotype characterised by reduced levels of Apo E protein in ε4 carriers. Third, some proteins (SGOT, MCP-1, and IL6-r) were found to be related to a longitudinal change in MMSE score over a 4 year period. Although the statistical effects associated with this finding were mild and no result passed multiple comparison correction, further studies will determine whether they may serve as important prognostic factors related to the rate of cognitive decline. More importantly, we showed that reducing the RBM panel to a subset of 24 CSF proteins complemented existing AD biomarkers for AD detection and MCI to AD conversion prediction.

Our findings were in agreement with some previous studies identifying a panel of candidate proteins associated with AD [15,21,39]. In particular, our first finding showed that several proteins were associated with brain atrophy and CSF biomarkers, however, levels of CgA and FABP emerged as the most consistently present across most our comparisons. Although the effects associated with these findings were mild, previous studies have linked these candidates to AD pathophysiology [40,41]. For instance, we found elevated levels of FABP protein to be significantly related to neuroimaging SPARE-AD score which is in agreement with previous findings reporting elevated levels of FABP protein in AD and prodromal MCI subjects [15,21,42]. Increased levels of CgA protein related to hippocampal and entorhinal volume has also been previously linked to early synaptic dysfunction in AD [40], reduced microglial regulation of synaptic function [43], and Aβ_1–42_ metabolism in CN individuals [44].

We also found a unique biochemical profile of CSF proteins was associated with different ApoE gene polymorphisms. For instance, ApoE protein and IL-3 levels were reduced in ε4 carriers, whilst MIF protein levels were elevated. Previous studies have also reported a peripheral CSF signature associated with ApoE genotype [44] and similar findings have also been observed in blood plasma [45]. Moreover, many of the CSF candidates previously described in the literature (e.g. FABP, FGF-4, IL-8, AGRP, ANG-2, and Osteopontin) also showed mild associations with ApoE genotype, suggesting that the biological variability of proteins identified in AD cases may also be in part driven by genotype status.

For differentiating between AD and CN individuals we found that a subset of proteins (n = 24) from the RBM multiplex panel improved the accuracy and performance of models but was unable to achieve a better accuracy when regional MRI measures and CSF biomarkers were combined. Despite this, the combination of regional MRI measures, CSF biomarkers (Aβ_1–42_, T-tau and P-tau) and the CSF RFE subset achieved an accuracy of 91.5%. In this subset, four proteins namely CgA, FABP, MMP-2, and PPP were the strongest predictors for distinguishing AD from CN individuals.

This is in agreement with previous studies showing that CSF candidates identified using immunoassay panel technology can complement CSF biomarkers of AD for the earlier detection of AD [21,46]. Recent neuroimaging-proteomic studies have also shown several proteins to be associated with longitudinal rates of brain atrophy [39], as well as whole brain atrophy [47]. To our knowledge this is the first study to test whether CSF proteins from an immunoassay panel can complement CSF biomarkers and regional MRI measures for disease classification and prediction. Previous studies have suggested that the use of conventional imaging, such as MRI, combined with biomarkers from different modalities may be complimentary to the early and specific diagnosis of AD [48,49]. Several studies have reported that this combined approach improved AD disease classification [10,36] and future MCI to AD conversion prediction [9,50].

For MCI to AD conversion prediction, the CSF RFE subset, CSF biomarker and regional MRI measures model also gave the best results and outperformed all other models. The model was particularly sensitive for correctly predicting MCI-c with an AD-like phenotype (Fig 4a) suggesting that our panel of 24 proteins may also have the prognostic potential to detect prodromal AD. However, the model failed to correctly detect MCI-nc, with a large proportion of subjects being predicted as AD-like, suggesting that the model lacked specificity. Several previous studies on MCI to AD conversion prediction have also noted the heterogeneity of the MCI-nc group using similar high dimensional pattern classification algorithms [10,51]. It is anticipated that many MCI-nc will convert to AD in the near future. Although future studies with longer follow up times will refine our estimates of specificity, the ability to detect MCI-nc many years prior to clinical diagnosis could provide useful tools for an earlier diagnosis.

Despite some promising results, there exist a number of limitations to our findings. Firstly, although we identified a number of CSF proteins showing promise in AD detection and MCI to AD conversion prediction our results are somewhat limited by the inability to validate these candidates in an independent cohort. Therefore future studies are warranted to further explore the prognostic potential of the candidates identified here in other well-characterised prospective cohorts. Nonetheless, we do show that the panel of CSF proteins for detecting AD also have a good prognostic potential for detecting AD in the prodromal or amnestic MCI stage. Secondly, CSF proteins identified in this study were from a multiplex panel of proteins known to be associated with microglial activity and synaptic function. It may be likely that an alternative set of CSF proteins unrelated to these processes could also show strong effects in detecting AD and predicting MCI to AD conversion.

In summary, the relation of CSF proteins to key neuroimaging phenotypes and traditional CSF biomarkers provides some evidence of their importance in reflecting early neuropathological changes in AD pathogenesis. Combining a subset of proteins (n = 24) from the RBM multiplex panel with established biomarkers in AD provides further evidence to implicate the role of peripheral CSF proteins for improving the accuracy and prognostic ability of biomarkers for disease classification and progression. Future studies are warranted to further validate our findings with more generalisability in other well-characterised independent cohorts.

## Supporting Information

S1 Table Complete list of analytes from the RBM multiplex panel used for analysis. (DOCX)

S2 Table Complete list of regional MRI measures from the Freesurfer image analysis pipeline used for analysis. (DOCX)

S3 Table Baseline CSF proteins that were significantly associated with regional MRI measures, SPARE-AD score or CSF biomarkers in AD patients and MCI subjects (n = 207). (DOCX)

## References

[1] Jack CR, Knopman DS, Jagust WJ, Shaw LM, Aisen PS, Weiner MW, et al Hypothetical model of dynamic biomarkers of the Alzheimer’s pathological cascade. Lancet Neurol. 2010;9: 119–28. 20083042 10.1016/S1474-4422(09)70299-6 PMC2819840

[2] Villemagne VL, Burnham S, Bourgeat P, Brown B, Ellis K a, Salvado O, et al Amyloid β deposition, neurodegeneration, and cognitive decline in sporadic Alzheimer’s disease: a prospective cohort study. Lancet Neurol. 2013;12: 357–67. 23477989 10.1016/S1474-4422(13)70044-9

[3] Braak H, Braak E. Neuropathological stageing of Alzheimer-related changes. Acta Neuropathol. Springer; 1991;82: 239–259. 10.1007/BF00308809 1759558

[4] Jack CR, Wiste HJ, Weigand SD, Rocca WA, Knopman DS, Mielke MM, et al Age-specific population frequencies of cerebral β-amyloidosis and neurodegeneration among people with normal cognitive function aged 50–89 years: a cross-sectional study. Lancet Neurol. 2014;13: 997–1005. 25201514 10.1016/S1474-4422(14)70194-2 PMC4324499

[5] Fox NC, Warrington EK, Freeborough PA, Hartikainen P, Kennedy AM, Stevens JM, et al Presymptomatic hippocampal atrophy in Alzheimer’s disease. A longitudinal MRI study. Brain. OXFORD UNIV PRESS; 1996;119: 2001–2007. 10.1093/brain/119.6.2001 9010004

[6] Desikan RS, Cabral HJ, Hess CP, Dillon WP, Glastonbury CM, Weiner MW, et al Automated MRI measures identify individuals with mild cognitive impairment and Alzheimer’s disease. Brain. Oxford University Press; 2009;132: 2048–2057. 10.1093/brain/awp123 PMC2714061 19460794

[7] Blennow K, Hampel H. CSF markers for incipient Alzheimer’s disease. Lancet. 2003;2: 605–613. 14505582 10.1016/s1474-4422(03)00530-1

[8] Shaw LM, Korecka M, Clark CM, Lee VM-Y, Trojanowski JQ. Biomarkers of neurodegeneration for diagnosis and monitoring therapeutics. Nat Rev Drug Discov. 2007;6: 295–303. 17347655 10.1038/nrd2176

[9] Davatzikos C, Bhatt P, Shaw LM, Batmanghelich KN, Trojanowski JQ. Prediction of MCI to AD conversion, via MRI, CSF biomarkers, and pattern classification. Neurobiol Aging. Elsevier Inc.; 2010; 10.1016/j.neurobiolaging.2010.05.023 PMC2951483 20594615

[10] Westman E, Muehlboeck J-S, Simmons A. Combining MRI and CSF measures for classification of Alzheimer’s disease and prediction of mild cognitive impairment conversion. Neuroimage. Elsevier B.V.; 2012;62: 229–38. 10.1016/j.neuroimage.2012.04.056 22580170

[11] Mattsson N, Zetterberg H, Hansson O, Andreasen N, Parnetti L, Jonsson M, et al CSF biomarkers and incipient Alzheimer disease in patients with mild cognitive impairment. JAMA. 2009;302: 385–93. 10.1001/jama.2009.1064 19622817

[12] Fagan AM, Perrin RJ. Upcoming candidate cerebrospinal fluid biomarkers of Alzheimer’s disease. Biomark Med. 2012;6: 455–476. 10.2217/bmm.12.42 22917147 PMC3477809

[13] Guerreiro R, Wojtas A, Bras J, Carrasquillo M, Rogaeva E, Majounie E, et al TREM2 variants in Alzheimer’s disease. N Engl J Med. 2013;368: 117–27. 10.1056/NEJMoa1211851 23150934 PMC3631573

[14] Zhang J, Goodlett DR, Peskind ER, Quinn JF, Zhou Y, Wang Q, et al Quantitative proteomic analysis of age-related changes in human cerebrospinal fluid. Neurobiol Aging. 2005;26: 207–27. 15582749 10.1016/j.neurobiolaging.2004.03.012

[15] Hu WT, Chen-Plotkin A, Arnold SE, Grossman M, Clark CM, Shaw LM, et al Novel CSF biomarkers for Alzheimer’s disease and mild cognitive impairment. Acta Neuropathol. 2010;119: 669–78. 10.1007/s00401-010-0667-0 20232070 PMC2880811

[16] Craig-Schapiro R, Perrin RJ, Roe CM, Xiong C, Carter D, Cairns NJ, et al YKL-40: a novel prognostic fluid biomarker for preclinical Alzheimer’s disease. Biol Psychiatry. Elsevier Inc.; 2010;68: 903–12. 10.1016/j.biopsych.2010.08.025 PMC3011944 21035623

[17] Weiner MW, Aisen PS, Jack CR, Jagust WJ, Trojanowski JQ, Shaw L, et al The Alzheimer’s disease neuroimaging initiative: progress report and future plans. Alzheimers Dement. Elsevier Ltd; 2010;6: 202–11. 10.1016/j.jalz.2010.03.007 PMC2927112 20451868

[18] Shaw LM, Vanderstichele H, Knapik-Czajka M, Clark CM, Aisen PS, Petersen RC, et al Cerebrospinal fluid biomarker signature in Alzheimer’s disease neuroimaging initiative subjects. Ann Neurol. 2009;65: 403–413. 10.1002/ana.21610 19296504 PMC2696350

[19] Shaw LM, Vanderstichele H, Knapik-Czajka M, Figurski M, Coart E, Blennow K, et al Qualification of the analytical and clinical performance of CSF biomarker analyses in ADNI. Acta Neuropathol. 2011;121: 597–609. 10.1007/s00401-011-0808-0 21311900 PMC3175107

[20] Trojanowski JQ, Vandeerstichele H, Korecka M, Clark CM, Aisen PS, Petersen RC, et al Update on the biomarker core of the Alzheimer’s Disease Neuroimaging Initiative subjects. Alzheimer’s Dement. Elsevier Ltd; 2010;6: 230–238. 10.1016/j.jalz.2010.03.008 PMC2867838 20451871

[21] Craig-Schapiro R, Kuhn M, Xiong C, Pickering EH, Liu J, Misko TP, et al Multiplexed immunoassay panel identifies novel CSF biomarkers for Alzheimer’s disease diagnosis and prognosis. PLoS One. 2011;6: e18850 10.1371/journal.pone.0018850 21526197 PMC3079734

[22] Hu WT, Holtzman DM, Fagan AM, Shaw LM, Perrin R, Arnold SE, et al Plasma multianalyte profiling in mild cognitive impairment and Alzheimer disease. Neurology. 2012;79: 897–905. 22855860 10.1212/WNL.0b013e318266fa70 PMC3425844

[23] ADNI cerebrospinal fliud aliquot inventory description report [Internet]. Available: ADNI_CSF_Aliquot_Inventory_Description_v08_16_12.pdf

[24] ADNI clinical procedures manual [Internet]. Dec. 10.1093/infdis/jis910

[25] ADNI: Alzheimer’s Disease Neuroimaging Initiative [Internet]. Available: http://www.adni-info.org/Scientists/ADNIStudyProcedures.aspx

[26] Jack CR, Bernstein MA, Fox NC, Thompson P, Alexander G, Harvey D, et al The Alzheimer’s disease neuroimaging initiative (ADNI): MRI methods. J Magn Reson Imaging. Wiley Online Library; 2008;27: 685–691. 10.1002/jmri.21049 PMC2544629 18302232

[27] Simmons A, Westman E, Muehlboeck S, Mecocci P, Vellas B, Tsolaki M, et al MRI measures of Alzheimer’s disease and the AddNeuroMed study. Ann N Y Acad Sci. 2009;1180: 47–55. 19906260 10.1111/j.1749-6632.2009.05063.x

[28] Simmons A, Westman E, Muehlboeck S, Mecocci P, Vellas B, Tsolaki M, et al The AddNeuroMed framework for multi-centre MRI assessment of Alzheimer’s disease: experience from the first 24 months. Int J Geriatr Psychiatry. 2011;26: 75–82. 10.1002/gps.2491 21157852

[29] Westman E, Simmons A, Zhang Y, Muehlboeck J-S, Tunnard C, Liu Y, et al Multivariate analysis of MRI data for Alzheimer’s disease, mild cognitive impairment and healthy controls. Neuroimage. Elsevier Inc.; 2011;54: 1178–87. 10.1016/j.neuroimage.2010.08.044 20800095

[30] Westman E, Simmons A, Muehlboeck J-S, Mecocci P, Vellas B, Tsolaki M, et al AddNeuroMed and ADNI: similar patterns of Alzheimer’s atrophy and automated MRI classification accuracy in Europe and North America. Neuroimage. Elsevier Inc.; 2011;58: 818–28. 10.1016/j.neuroimage.2011.06.065 21763442

[31] Westman E, Aguilar C, Muehlboeck J-S, Simmons A. Regional magnetic resonance imaging measures for multivariate analysis in Alzheimer’s disease and mild cognitive impairment. Brain Topogr. 2013;26: 9–23. 10.1007/s10548-012-0246-x 22890700 PMC3536978

[32] Fan Y, Batmanghelich N, Clark CM, Davatzikos C. Spatial patterns of brain atrophy in MCI patients, identified via high-dimensional pattern classification, predict subsequent cognitive decline. Neuroimage. 2008;39: 1731–1743. 18053747 10.1016/j.neuroimage.2007.10.031 PMC2861339

[33] Davatzikos C, Xu F, An Y, Fan Y, Resnick SM. Longitudinal progression of Alzheimer’s-like patterns of atrophy in normal older adults: the SPARE-AD index. Brain. 2009;132: 2026–35. 10.1093/brain/awp091 19416949 PMC2714059

[34] Chang C, Lin C-J. LIBSVM: A Library for Support Vector Machines. ACM Trans Intell Syst Technol. 2011;2: 1–27.

[35] Hsu C, Chang C, Lin C. A Practical Guide to Support Vector Classification. Bioinformatics. Citeseer; 2010;1: 1–16.

[36] Zhang D, Wang Y, Zhou L, Yuan H, Shen D. Multimodal classification of Alzheimer’s disease and mild cognitive impairment. Neuroimage. Elsevier Inc.; 2011;55: 856–867. 10.1016/j.neuroimage.2011.01.008 PMC3057360 21236349

[37] Schölkopf B, Smola AJ. Learning with Kernels Dietterich T, editor. Kybernetik. MIT Press; 2002.

[38] Aguilar C, Westman E, Muehlboeck J-S, Mecocci P, Vellas B, Tsolaki M, et al Different multivariate techniques for automated classification of MRI data in Alzheimer’s disease and mild cognitive impairment. Psychiatry Res. Elsevier; 2013;212: 89–98. 10.1016/j.pscychresns.2012.11.005 23541334

[39] Mattsson N, Insel P, Nosheny R, Trojanowski JQ, Shaw LM, Jack CR, et al Effects of cerebrospinal fluid proteins on brain atrophy rates in cognitively healthy older adults. Neurobiol Aging. Elsevier Ltd; 2014;35: 614–22. 10.1016/j.neurobiolaging.2013.08.027 PMC3864623 24094581

[40] Blennow K, Davidsson P, Wallin A, Ekman R. Chromogranin A in cerebrospinal fluid: a biochemical marker for synaptic degeneration in Alzheimer’s disease? Dementia. 1995;6: 306–311. 8563783 10.1159/000106963

[41] Chiasserini D, Parnetti L, Andreasson U, Zetterberg H, Giannandrea D, Calabresi P, et al CSF levels of heart fatty acid binding protein are altered during early phases of Alzheimer’s disease. J Alzheimers Dis. 2010;22: 1281–8. 10.3233/JAD-2010-101293 20930282

[42] Olsson B, Hertze J, Ohlsson M, Nägga K, Höglund K, Basun H, et al Cerebrospinal fluid levels of heart fatty acid binding protein are elevated prodromally in Alzheimer’s disease and vascular dementia. J Alzheimers Dis. 2013;34: 673–9. 10.3233/JAD-121384 23254629

[43] Taylor DL, Diemel LT, Cuzner ML, Pocock JM. Activation of group II metabotropic glutamate receptors underlies microglial reactivity and neurotoxicity following stimulation with chromogranin A, a peptide up-regulated in Alzheimer’s disease. J Neurochem. 2002;82: 1179–1191. 12358765 10.1046/j.1471-4159.2002.01062.x

[44] Mattsson N, Insel P, Nosheny R, Zetterberg H, Trojanowski JQ, Shaw LM, et al CSF protein biomarkers predicting longitudinal reduction of CSF β-amyloid42 in cognitively healthy elders. Transl Psychiatry. 2013;3: e293 10.1038/tp.2013.69 23962923 PMC3756294

[45] Soares HD, Potter WZ, Pickering E, Kuhn M, Immermann FW, Shera DM, et al Plasma Biomarkers Associated With the Apolipoprotein E Genotype and Alzheimer Disease. Arch Neurol. 2012; 1–8. 10.1001/archneurol.2012.1070 PMC3683865 22801723

[46] Perrin RJ, Craig-Schapiro R, Malone JP, Shah AR, Gilmore P, Davis AE, et al Identification and validation of novel cerebrospinal fluid biomarkers for staging early Alzheimer’s disease. PLoS One. 2011;6: e16032 10.1371/journal.pone.0016032 21264269 PMC3020224

[47] Paterson RW, Bartlett JW, Blennow K, Fox NC, Shaw LM, Trojanowski JQ, et al Cerebrospinal fluid markers including trefoil factor 3 are associated with neurodegeneration in amyloid-positive individuals. Transl Psychiatry. Nature Publishing Group; 2014;4: 419. 10.1038/tp.2014.58 PMC4119225 25072324

[48] Vemuri P, Wiste HJ, Weigand SD, Shaw LM, Trojanowski JQ, Weiner MW, et al MRI and CSF biomarkers in normal, MCI, and AD subjects: predicting future clinical change. Neurology. 28 7 2009 73: 294–301. 10.1212/WNL.0b013e3181af79fb 19636049 PMC2715214

[49] Apostolova LG, Hwang KS, Andrawis JP, Green AE, Babakchanian S, Morra JH, et al 3D PIB and CSF biomarker associations with hippocampal atrophy in ADNI subjects. Neurobiol Aging. Elsevier Inc.; 2010;31: 1284–1303. 10.1016/j.neurobiolaging.2010.05.003 PMC3051831 20538372

[50] Cui Y, Liu B, Luo S, Zhen X, Fan M, Liu T, et al Identification of Conversion from Mild Cognitive Impairment to Alzheimer’s Disease Using Multivariate Predictors. PLoS One. Public Library of Science; 2011;6: e21896. 10.1371/journal.pone.0021896 PMC3140993 21814561

[51] Misra C, Fan Y, Davatzikos C. Baseline and longitudinal patterns of brain atrophy in MCI patients, and their use in prediction of short-term conversion to AD: results from ADNI. Neuroimage. Elsevier Inc.; 2009;44: 1415–22. 10.1016/j.neuroimage.2008.10.031 PMC2648825 19027862

