# Supplementary material for: A Subset of Cerebrospinal Fluid Proteins from a Multi-Analyte Panel Associated with Brain Atrophy, Disease Classification and Prediction in Alzheimer’s Disease
Source: PLoS One. 2015 Aug 18;10(8):e0134368. doi: 10.1371/journal.pone.0134368 (PMC4540455; doi:10.1371/journal.pone.0134368)
Supplement: S1 Table — (DOCX) [file pone.0134368.s001.docx]

| **Table S1**. List of RBM analytes from the multiplex panel that were available for analysis (*n*=89).  Tr indicates that the analyte was transformed to approximate a normal distribution following inspection by Box-Cox methods.  Accession numbers for each of these analytes and details of the assays are listed on [www.rulesbasedmedicine.com](http://www.rulesbasedmedicine.com).   \| **RBM analyte** \| **RBM analyte** \| \| --- \| --- \| \| Alpha-1-Antitrypsin (AAT) \| Tr Alpha-2-Macroglobulin (A2Macro) \| \| Alpha-1-Microglobulin (A1Micro) \| Tr Apolipoprotein A-I (Apo A-I) \| \| Angiopoietin-2 (ANG-2) \| Tr Apolipoprotein C-III (Apo C-III) \| \| Angiotensin-Converting Enzyme (ACE) \| Tr Apolipoprotein E (Apo E) \| \| Apolipoprotein D (Apo D) \| Tr Apolipoprotein(a) (Lp(a)) \| \| Apolipoprotein H (Apo H) \| Tr AXL Receptor Tyrosine Kinase (AXL) \| \| CD 40 antigen (CD40) \| Tr Beta-2-Microglobulin (B2M) \| \| Chromogranin-A (CgA) \| Tr Calcitonin \| \| Clusterin (CLU) \| Tr Cancer Antigen 19-9 (CA-19-9) \| \| Cystatin-C \| Tr Chemokine CC-4 (HCC-4) \| \| Ferritin (FRTN) \| Tr Complement C3 (C3) \| \| Immunoglobulin A (IgA) \| Tr Cortisol \| \| Interleukin-6 receptor (IL-6r) \| Tr C-Reactive Protein (CRP) \| \| Macrophage Colony-Stimulating Factor 1 (M-CSF) \| Tr Endothelin-1 (ET-1) \| \| Matrix Metalloproteinase-3 (MMP-3) \| Tr Fas Ligand (FasL) \| \| Monocyte Chemotactic Protein 1 (MCP-1) \| Tr Fatty Acid-Binding Protein, heart (FABP, heart) \| \| Neutrophil Gelatinase-Associated Lipocalin (NGAL) \| Tr Fibrinogen \| \| N-terminal prohormone of brain natriuretic peptide (NT proBNP) \| Tr Fibroblast Growth Factor 4 (FGF-4) \| \| Osteopontin \| Tr Follicle-Stimulating Hormone (FSH) \| \| Pancreatic Polypeptide (PPP) \| Tr Heparin-Binding EGF-Like Growth Factor (HB-EGF) \| \| Serum Glutamic Oxaloacetic Transaminase (SGOT) \| Tr Hepatocyte Growth Factor (HGF) \| \| Sortilin \| Tr Insulin-like Growth Factor-Binding Protein 2 (IGFBP-2) \| \| Tissue Factor (TF) \| Tr Intercellular Adhesion Molecule 1 (ICAM-1) \| \| TNF-Related Apoptosis-Inducing Ligand Receptor 3 (TRAIL-R3) \| Tr Interferon gamma Induced Protein 10 (IP-10) \| \| Tr Adiponectin \| Tr Interleukin-16 (IL-16) \| \| Tr Agouti-Related Protein (AGRP) \| Tr Interleukin-25 (IL-25) \| \| Tr Interleukin-3 (IL-3) \| Tr T Lymphocyte-Secreted Protein I-309 (I-309) \| \| Tr Interleukin-8 (IL-8) \| Tr T-Cell-Specific Protein RANTES (RANTES) \| \| Tr Lectin-Like Oxidized LDL Receptor 1 (LOX-1) \| Tr Thrombomodulin (TM) \| \| Tr Leptin \| Tr Thyroxine-Binding Globulin (TBG) \| \| Tr Macrophage Inflammatory Protein-1 beta (MIP-1 beta) \| Tr Tissue Inhibitor of Metalloproteinases 1 (TIMP-1) \| \| Tr Macrophage Migration Inhibitory Factor (MIF) \| Tr Transforming Growth Factor alpha (TGF-alpha) \| \| Tr Matrix Metalloproteinase-2 (MMP-2) \| Tr Trefoil Factor 3 (TFF3) \| \| Tr Monocyte Chemotactic Protein 2 (MCP-2) \| Tr Tumor Necrosis Factor Receptor 2 (TNFR2) \| \| Tr Monokine Induced by Gamma Interferon (MIG) \| Tr Vascular Cell Adhesion Molecule-1 (VCAM-1) \| \| Tr Myoglobin \| Tr von Willebrand Factor (vWF) \| \| Tr Placenta Growth Factor (PLGF) \| Vascular Endothelial Growth Factor (VEGF) \| \| Tr Plasminogen Activator Inhibitor 1 (PAI-1) \|  \| \| Tr Pregnancy-Associated Plasma Protein A (PAPP-A) \|  \| \| Tr Prolactin \|  \| \| Tr Prostatic Acid Phosphatase (PAP) \|  \| \| Tr Resistin \|  \| \| Tr S100 calcium-binding protein B (S100-B) \|  \| \| Tr Serum Amyloid P-Component (SAP) \|  \| \| Tr Sex Hormone-Binding Globulin (SHBG) \|  \| \| Tr Stem Cell Factor (SCF) \|  \| |
| --- | --- | --- | --- | --- | --- | --- | --- | --- | --- | --- | --- | --- | --- | --- | --- | --- | --- | --- | --- | --- | --- | --- | --- | --- | --- | --- | --- | --- | --- | --- | --- | --- | --- | --- | --- | --- | --- | --- | --- | --- | --- | --- | --- | --- | --- | --- | --- | --- | --- | --- | --- | --- | --- | --- | --- | --- | --- | --- | --- | --- | --- | --- | --- | --- | --- | --- | --- | --- | --- | --- | --- | --- | --- | --- | --- | --- | --- | --- | --- | --- | --- | --- | --- | --- | --- | --- | --- | --- | --- | --- | --- | --- | --- | --- |
