# Supplementary material for: A Subset of Cerebrospinal Fluid Proteins from a Multi-Analyte Panel Associated with Brain Atrophy, Disease Classification and Prediction in Alzheimer’s Disease
Source: PLoS One. 2015 Aug 18;10(8):e0134368. doi: 10.1371/journal.pone.0134368 (PMC4540455; doi:10.1371/journal.pone.0134368)
Supplement: S2 Table — (DOCX) [file pone.0134368.s002.docx]

| **Table S2:** List of Regional MRI measures used in the support vector machine algorithm. Note: Both left and right hemisphere regions were used in the analysis.   \| **Cortical Thickness measures (n=34)** \| **Volumetric measures (n=23)** \| \| --- \| --- \| \| Banks of superior temporal sulcus \| Third ventricle \| \| Caudal anterior cingulate \| Fourth ventricle \| \| Caudal middle frontal gyrus \| Brainstem \| \| Cuneus cortex \| Corpus callosum anterior \| \| Entorhinal cortex \| Corpus callosum central \| \| Fusiform gyrus \| Corpus callosum midanterior \| \| Inferior parietal cortex \| Corpus callosum midposterior \| \| Inferior temporal gyrus \| Corpus callosum posterior \| \| Isthmus of cingulate cortex \| CSF \| \| Lateral occipital cortex \| Accumbens \| \| Lateral orbitofrontal cortex \| Amygdala \| \| Lingual gyrus \| Caudate \| \| Medial orbitofrontal cortex \| Cerebellum cortex \| \| Middle temporal gyrus \| Cerebellum white matter \| \| Parahippocampal gyrus \| Hippocampus \| \| Paracentral sulcus \| Inferior lateral ventricle \| \| Frontal operculum \| Putamen \| \| Orbital operculum \| Cerebral cortex \| \| Triangular part of inferior frontal gyrus \| Cerebral white matter \| \| Pericalcarine cortex \| Lateral ventricle \| \| Postcentral gyrus \| Pallidum \| \| Posterior cingulate cortex \| Thalamus proper \| \| Precentral gyrus \| Ventral DC \| \| Precuneus cortex \|  \| \| Rostral anterior cingulate cortex \|  \| \| Rostral middle frontal gyrus \|  \| \| Superior frontal gyrus \|  \| \| Superior parietal gyrus \|  \| \| Superior temporal gyrus \|  \| \| Supramarginal gyrus \|  \| \| Frontal pole \|  \| \| Temporal pole \|  \| \| Transverse temporal cortex \|  \| \| Insular Banks of superior temporal sulcus \|  \| |
| --- | --- | --- | --- | --- | --- | --- | --- | --- | --- | --- | --- | --- | --- | --- | --- | --- | --- | --- | --- | --- | --- | --- | --- | --- | --- | --- | --- | --- | --- | --- | --- | --- | --- | --- | --- | --- | --- | --- | --- | --- | --- | --- | --- | --- | --- | --- | --- | --- | --- | --- | --- | --- | --- | --- | --- | --- | --- | --- | --- | --- | --- | --- | --- | --- | --- | --- | --- | --- | --- | --- |
