# Supplementary material for: A Subset of Cerebrospinal Fluid Proteins from a Multi-Analyte Panel Associated with Brain Atrophy, Disease Classification and Prediction in Alzheimer’s Disease
Source: PLoS One. 2015 Aug 18;10(8):e0134368. doi: 10.1371/journal.pone.0134368 (PMC4540455; doi:10.1371/journal.pone.0134368)
Supplement: S3 Table — (DOCX) [file pone.0134368.s003.docx]

| \| **RBM analyte** \| **Spearman’s Rank Correlation** \| **Uncorrected *P*-value** \| **False-Discovery Rate Corrected *P*-value** \| \| --- \| --- \| --- \| --- \| \| *Hippocampal Volume* \| \| \| \| \| **Chromogranin A (CgA) [ng/mL]**^a^ \| **0.293** \| **<0.0001** \| **<0.001** \| \| Tissue Factor (TF) [ng/mL]^a^ \| 0.212 \| 0.002 \| 0.176 \| \| Angiotensin-Converting Enzyme (ACE) [ng/mL]^a^ \| 0.204 \| 0.003 \| 0.272 \| \| CD 40 antigen (CD40) [ng/mL] \| 0.198 \| 0.004 \| 0.363 \| \| Stem Cell Factor (SCF) [pg/mL] \| 0.197 \| 0.005 \| 0.378 \| \| Vascular Endothelial Growth Factor (VEGF) [ng/mL]^a^ \| 0.193 \| 0.006 \| 0.457 \| \| Cystatin – C [ng/mL]^a^ \| -0.171 \| 0.014 \| 1.000 \| \| Monokine Induced by Gamma Interferon (MI) [pg/mL] \| 0.144 \| 0.040 \| 1.000 \| \| AXL Receptor Tyrosine Kinase (AXL) [ng/mL] \| -0.143 \| 0.040 \| 1.000 \| \| Placenta Growth Factor (PLGF) [pg/mL] \| 0.143 \| 0.041 \| 1.000 \| \| *Entorhinal Volume* \| \| \| \| \| **Chromogranin A (CgA) [ng/mL]** \| **0.265** \| **0.000** \| **0.008** \| \| Vascular Endothelial Growth Factor (VEGF) [ng/mL] \| 0.207 \| 0.003 \| 0.232 \| \| Heparin-Binding EGF-Like Growth Factor (HB-EGF) [pg/mL] \| 0.192 \| 0.006 \| 0.470 \| \| Plasminogen Activator Inhibitor 1 (PAI-1) [ng/mL] \| 0.168 \| 0.016 \| 1.000 \| \| Lectin-Like Oxidized LDL Receptor 1 (LOX-1) [ng/mL] \| -0.166 \| 0.017 \| 1.000 \| \| Cystatin – C [ng/mL] \| -0.166 \| 0.018 \| 1.000 \| \| Macrophage Colony-Stimulating Factor 1 (M-CSF) [ng/mL] \| 0.158 \| 0.023 \| 1.000 \| \| Tissue Factor (TF) [ng/mL] \| 0.149 \| 0.033 \| 1.000 \| \| Angiotensin-Converting Enzyme (ACE) [ng/mL] \| 0.146 \| 0.037 \| 1.000 \| \| *SPARE-AD Score* \| \| \| \| \| **Fatty Acid-Binding Protein, heart (FABP, heart)** \| **0.236** \| **0.000** \| **0.003** \| \| Chromogranin A (CgA) [ng/mL] \| -0.163 \| 0.005 \| 0.416 \| \| CD 40 antigen (CD40) [ng/mL] \| -0.162 \| 0.005 \| 0.447 \| \| N-terminal prohormone of brain natriuretic peptide  (NT proBNP) [pg/mL] \| 0.154 \| 0.008 \| 0.662 \| \| Macrophage Migration Inhibitory Factor (MIF) [ng/mL] \| -0.127 \| 0.029 \| 1.000 \| \| Pancreatic Polypeptide (PPP) [pg/mL] \| 0.120 \| 0.041 \| 1.000 \| \| Osteopontin [ng/mL] \| 0.118 \| 0.043 \| 1.000 \| \| Interleukin-6 receptor (IL-6r) [ng/mL] \| -0.113 \| 0.052 \| 1.000 \| \| *Aβ 1-42* \| \| \| \| \| Hepatocyte Growth Factor (HGF) [ng/mL] \| -0.196 \| 0.005 \| 0.391 \| \| Osteopontin [ng/mL] \| -0.193 \| 0.005 \| 0.444 \| \| Immunoglobulin A (IgA) [mg/mL] \| 0.160 \| 0.022 \| 1.000 \| \| Sex Hormone-Binding Globulin (SHBG) [nmol/L] \| -0.139 \| 0.047 \| 1.000 \| \| C-Reactive Protein (CRP) [ug/mL] \| 0.138 \| 0.049 \| 1.000 \| \| Chromogranin A (CgA) [ng/mL] \| -0.136 \| 0.052 \| 1.000 \| \| *P-Tau181* \| \| \| \| \| **Fatty Acid-Binding Protein, heart (FABP, heart)** \| **0.556** \| **<0.001** \| **<0.001** \| \| **Tissue Factor (TF) [ng/mL]** \| **0.479** \| **<0.001** \| **<0.001** \| \| **Cystatin – C [ng/mL]** \| **-0.479** \| **<0.001** \| **<0.001** \| \| **Chromogranin A (CgA) [ng/mL]** \| **0.431** \| **<0.001** \| **<0.001** \| \| **Hepatocyte Growth Factor (HGF) [ng/mL]** \| **0.410** \| **<0.001** \| **<0.001** \| \| **Angiopoietin-2 (ANG-2) [ng/mL]** \| **0.403** \| **<0.001** \| **<0.001** \| \| **Lectin-Like Oxidized LDL Receptor 1 (LOX-1) [ng/mL]** \| **-0.386** \| **<0.001** \| **<0.001** \| \| **Tumor Necrosis Factor Receptor 2 (TNFR2) [ng/mL]** \| **-0.380** \| **<0.001** \| **<0.001** \| \| **Apolipoprotein E (Apo E) [ug/mL]** \| **0.373** \| **<0.001** \| **<0.001** \| \| **Matrix Metalloproteinase-3 (MMP-3) [ng/mL]** \| **0.351** \| **<0.001** \| **<0.001** \| \| **Angiotensin-Converting Enzyme (ACE) [ng/mL]** \| **0.349** \| **<0.001** \| **<0.001** \| \| **Heparin-Binding EGF-Like Growth Factor (HB-EGF) [pg/mL]** \| **0.338** \| **<0.001** \| **<0.001** \| \| **Transforming Growth Factor alpha (TGF-alpha) [pg/mL]** \| **0.321** \| **<0.001** \| **<0.001** \| \| **Vascular Endothelial Growth Factor (VEGF) [ng/mL]** \| **0.315** \| **<0.001** \| **<0.001** \| \| **Macrophage Colony-Stimulating Factor 1 (M-CSF) [ng/mL]** \| **0.314** \| **<0.001** \| **<0.001** \| \| **Beta-2-Microglobulin (B2M) [ug/mL]** \| **-0.311** \| **<0.001** \| **<0.001** \| \| **CD 40 antigen (CD40) [ng/mL]** \| **0.310** \| **<0.001** \| **<0.001** \| \| **Stem Cell Factor (SCF) [pg/mL]** \| **0.301** \| **<0.001** \| **<0.001** \| \| **Osteopontin [ng/mL]** \| **0.294** \| **<0.001** \| **0.001** \| \| **AXL Receptor Tyrosine Kinase (AXL) [ng/mL]** \| **-0.290** \| **<0.001** \| **0.002** \| \| **Clusterin (CLU) [ug/mL]** \| **0.288** \| **<0.001** \| **0.002** \| \| **Insulin-like Growth Factor-Binding Protein 2 (IGFBP-2) [ng/mL]** \| **-0.281** \| **<0.001** \| **0.003** \| \| **Ferritin (FRTN) [ng/mL]** \| **0.277** \| **<0.001** \| **0.004** \| \| **Trefoil Factor 3 (TFF3) [ug/mL]** \| **-0.271** \| **<0.001** \| **0.006** \| \| **Fibroblast Growth Factor 4 (FGF-4) [pg/mL]** \| **-0.268** \| **<0.001** \| **0.007** \| \| **von Willebrand Factor (vWF) [ug/mL]** \| **-0.251** \| **<0.001** \| **0.020** \| \| **Interleukin-6 receptor (IL-6r) [ng/mL]** \| **0.249** \| **<0.001** \| **0.024** \| \| **S100 calcium-binding protein B (S100-B) [ng/mL]** \| **0.247** \| **<0.001** \| **0.026** \| \| **Vascular Cell Adhesion Molecule-1 (VCAM-1) [ng/mL]** \| **-0.234** \| **<0.001** \| **0.056** \| \| **TNF-Related Apoptosis-Inducing Ligand Receptor 3 (TRAIL-R3) [ng/mL]** \| **0.227** \| **<0.001** \| **0.080** \| \| Alpha-2-Macroglobulin (A2Macro) [mg/mL] \| -0.215 \| 0.002 \| 0.152 \| \| Sortilin [ng/mL] \| 0.210 \| 0.002 \| 0.202 \| \| T-Cell-Specific Protein RANTES (RANTES) [ng/mL] \| -0.184 \| 0.008 \| 0.662 \| \| Interleukin-3 (IL-3) [ng/mL] \| 0.179 \| 0.010 \| 0.850 \| \| Resistin [ng/mL] \| 0.163 \| 0.020 \| 1.000 \| \| Fas Ligand (FasL) [pg/mL] \| -0.161 \| 0.021 \| 1.000 \| \| Leptin [ng/mL] \| -0.160 \| 0.022 \| 1.000 \| \| Cancer Antigen 19-9 (CA-19-9) [U/mL] \| -0.158 \| 0.024 \| 1.000 \| \| N-terminal prohormone of brain natriuretic peptide  (NT proBNP) [pg/mL] \| 0.148 \| 0.034 \| 1.000 \| \| Follicle-Stimulating Hormone (FSH) [pg/mL] \| 0.141 \| 0.044 \| 1.000 \| \| *T-Tau* \| \| \| \| \| **Fatty Acid-Binding Protein, heart (FABP, heart)** \| **0.634** \| **<0.001** \| **<0.001** \| \| **Tissue Factor (TF) [ng/mL]** \| **0.590** \| **<0.001** \| **<0.001** \| \| **Cystatin – C [ng/mL]** \| **-0.582** \| **<0.001** \| **<0.001** \| \| **Chromogranin A (CgA) [ng/mL]** \| **0.535** \| **<0.001** \| **<0.001** \| \| **Lectin-Like Oxidized LDL Receptor 1 (LOX-1) [ng/mL]** \| **-0.512** \| **<0.001** \| **<0.001** \| \| **Osteopontin [ng/mL]** \| **0.478** \| **<0.001** \| **<0.001** \| \| **Apolipoprotein E (Apo E) [ug/mL]** \| **0.476** \| **<0.001** \| **<0.001** \| \| **Hepatocyte Growth Factor (HGF) [ng/mL]** \| **0.463** \| **<0.001** \| **<0.001** \| \| **Tumor Necrosis Factor Receptor 2 (TNFR2) [ng/mL]** \| **-0.461** \| **<0.001** \| **<0.001** \| \| **Vascular Endothelial Growth Factor (VEGF) [ng/mL]^a^** \| **0.452** \| **<0.001** \| **<0.001** \| \| **Angiotensin-Converting Enzyme (ACE) [ng/mL]** \| **0.447** \| **<0.001** \| **<0.001** \| \| **Matrix Metalloproteinase-3 (MMP-3) [ng/mL]** \| **0.436** \| **<0.001** \| **<0.001** \| \| **AXL Receptor Tyrosine Kinase (AXL) [ng/mL]** \| **-0.435** \| **<0.001** \| **<0.001** \| \| **Beta-2-Microglobulin (B2M) [ug/mL]** \| **-0.421** \| **<0.001** \| **<0.001** \| \| **Heparin-Binding EGF-Like Growth Factor (HB-EGF) [pg/mL]** \| **0.417** \| **<0.001** \| **<0.001** \| \| **Angiopoietin-2 (ANG-2) [ng/mL]** \| **0.400** \| **<0.001** \| **<0.001** \| \| **CD 40 antigen (CD40) [ng/mL]** \| **0.387** \| **<0.001** \| **<0.001** \| \| **Clusterin (CLU) [ug/mL]** \| **0.387** \| **<0.001** \| **<0.001** \| \| **Macrophage Colony-Stimulating Factor 1 (M-CSF) [ng/mL]** \| **0.386** \| **<0.001** \| **<0.001** \| \| **Transforming Growth Factor alpha (TGF-alpha) [pg/mL]** \| **0.377** \| **<0.001** \| **<0.001** \| \| **Stem Cell Factor (SCF) [pg/mL]** \| **0.363** \| **<0.001** \| **<0.001** \| \| **Ferritin (FRTN) [ng/mL]** \| **0.343** \| **<0.001** \| **<0.001** \| \| **S100 calcium-binding protein B (S100-B) [ng/mL]** \| **0.339** \| **<0.001** \| **<0.001** \| \| **Sortilin [ng/mL]** \| **0.337** \| **<0.001** \| **<0.001** \| \| **von Willebrand Factor (vWF) [ug/mL]** \| **-0.314** \| **<0.001** \| **<0.001** \| \| **Vascular Cell Adhesion Molecule-1 (VCAM-1) [ng/mL]** \| **-0.307** \| **<0.001** \| **<0.001** \| \| Interleukin-6 receptor (IL-6r) [ng/mL] \| 0.290 \| <0.001 \| 0.002 \| \| Trefoil Factor 3 (TFF3) [ug/mL] \| -0.269 \| <0.001 \| 0.006 \| \| Insulin-like Growth Factor-Binding Protein 2 (IGFBP-2) [ng/mL] \| -0.263 \| <0.001 \| 0.010 \| \| TNF-Related Apoptosis-Inducing Ligand Receptor 3 (TRAIL-R3) [ng/mL] \| 0.251 \| <0.001 \| 0.020 \| \| Alpha-2-Macroglobulin (A2Macro) [mg/mL] \| -0.248 \| <0.001 \| 0.024 \| \| Fibroblast Growth Factor 4 (FGF-4) [pg/mL] \| -0.248 \| <0.001 \| 0.024 \| \| Interleukin-3 (IL-3) [ng/mL] \| 0.201 \| 0.004 \| 0.309 \| \| N-terminal prohormone of brain natriuretic peptide  (NT proBNP) [pg/mL] \| 0.193 \| 0.005 \| 0.440 \| \| Leptin [ng/mL] \| -0.184 \| 0.008 \| 0.684 \| \| Monocyte Chemotactic Protein 1 (MCP-1) [pg/mL] \| 0.175 \| 0.012 \| 0.987 \| \| Matrix Metalloproteinase-2 (MMP-2) [ng/mL] \| 0.170 \| 0.015 \| 1.000 \| \| Cancer Antigen 19-9 (CA-19-9) [U/mL] \| -0.168 \| 0.016 \| 1.000 \| \| Intercellular Adhesion Molecule 1 (ICAM-1) [ng/mL] \| -0.159 \| 0.023 \| 1.000 \| \| Fibrinogen [mg/mL] \| -0.159 \| 0.023 \| 1.000 \| \| Follicle-Stimulating Hormone (FSH) [pg/mL] \| 0.136 \| 0.051 \| 1.000 \| \|  \|  \|  \|  \|   **Table S3**: CSF proteins from the multiplex panel associated with neuroimaging markers of brain atrophy, SPARE-AD score, and CSF biomarkers of AD. |
| --- | --- | --- | --- | --- | --- | --- | --- | --- | --- | --- | --- | --- | --- | --- | --- | --- | --- | --- | --- | --- | --- | --- | --- | --- | --- | --- | --- | --- | --- | --- | --- | --- | --- | --- | --- | --- | --- | --- | --- | --- | --- | --- | --- | --- | --- | --- | --- | --- | --- | --- | --- | --- | --- | --- | --- | --- | --- | --- | --- | --- | --- | --- | --- | --- | --- | --- | --- | --- | --- | --- | --- | --- | --- | --- | --- | --- | --- | --- | --- | --- | --- | --- | --- | --- | --- | --- | --- | --- | --- | --- | --- | --- | --- | --- | --- | --- | --- | --- | --- | --- | --- | --- | --- | --- | --- | --- | --- | --- | --- | --- | --- | --- | --- | --- | --- | --- | --- | --- | --- | --- | --- | --- | --- | --- | --- | --- | --- | --- | --- | --- | --- | --- | --- | --- | --- | --- | --- | --- | --- | --- | --- | --- | --- | --- | --- | --- | --- | --- | --- | --- | --- | --- | --- | --- | --- | --- | --- | --- | --- | --- | --- | --- | --- | --- | --- | --- | --- | --- | --- | --- | --- | --- | --- | --- | --- | --- | --- | --- | --- | --- | --- | --- | --- | --- | --- | --- | --- | --- | --- | --- | --- | --- | --- | --- | --- | --- | --- | --- | --- | --- | --- | --- | --- | --- | --- | --- | --- | --- | --- | --- | --- | --- | --- | --- | --- | --- | --- | --- | --- | --- | --- | --- | --- | --- | --- | --- | --- | --- | --- | --- | --- | --- | --- | --- | --- | --- | --- | --- | --- | --- | --- | --- | --- | --- | --- | --- | --- | --- | --- | --- | --- | --- | --- | --- | --- | --- | --- | --- | --- | --- | --- | --- | --- | --- | --- | --- | --- | --- | --- | --- | --- | --- | --- | --- | --- | --- | --- | --- | --- | --- | --- | --- | --- | --- | --- | --- | --- | --- | --- | --- | --- | --- | --- | --- | --- | --- | --- | --- | --- | --- | --- | --- | --- | --- | --- | --- | --- | --- | --- | --- | --- | --- | --- | --- | --- | --- | --- | --- | --- | --- | --- | --- | --- | --- | --- | --- | --- | --- | --- | --- | --- | --- | --- | --- | --- | --- | --- | --- | --- | --- | --- | --- | --- | --- | --- | --- | --- | --- | --- | --- | --- | --- | --- | --- | --- | --- | --- | --- | --- | --- | --- | --- | --- | --- | --- | --- | --- | --- | --- | --- | --- | --- | --- | --- | --- | --- | --- | --- | --- | --- | --- | --- | --- | --- | --- | --- | --- | --- | --- | --- | --- | --- | --- | --- | --- | --- | --- | --- | --- | --- | --- | --- | --- | --- | --- | --- | --- | --- | --- | --- | --- | --- | --- | --- | --- | --- | --- | --- | --- | --- | --- | --- | --- | --- | --- | --- | --- | --- | --- | --- | --- | --- | --- | --- | --- | --- | --- | --- | --- | --- | --- | --- | --- | --- | --- | --- | --- | --- | --- | --- | --- | --- | --- | --- | --- | --- | --- | --- | --- | --- | --- | --- | --- | --- | --- | --- | --- | --- | --- | --- | --- | --- | --- | --- | --- | --- | --- | --- | --- | --- | --- | --- | --- | --- | --- | --- | --- | --- |
